# Upregulation of Protein Tyrosine Phosphatase Type IVA Member 3 (PTP4A3/PRL-3) is Associated with Tumor Differentiation and a Poor Prognosis in Human Hepatocellular Carcinoma

**DOI:** 10.1245/s10434-012-2395-2

**Published:** 2012-10-13

**Authors:** Abudureheman Mayinuer, Mahmut Yasen, Kaoru Mogushi, Gulanbar Obulhasim, Maimaiti Xieraili, Arihiro Aihara, Shinji Tanaka, Hiroshi Mizushima, Hiroshi Tanaka, Shigeki Arii

**Affiliations:** 1 Department of Hepato-Biliary-Pancreatic Surgery, Tokyo Medical and Dental University, Tokyo, Japan; 2 Department of Surgery, Xinjiang Uyghur Tumor Hospital of Xinjiang Medical University, Xinjiang, China; 3 Department of Computational Biology and Bioinformatics, Tokyo Medical and Dental University, Tokyo, Japan

## Abstract

**Background:**

Protein tyrosine phosphatase type IVA member 3 (PTP4A3/PRL-3), a metastasis-associated phosphatase, plays multiple roles in cancer metastasis. We investigated PTP4A3/PRL-3 expression and its correlation with the clinicopathological features and prognosis in hepatocellular carcinoma (HCC).

**Methods:**

Gene expression profiles of PTP4A3/PRL-3 were obtained in poorly differentiated HCC tissues. The results were validated independently by TaqMan gene expression assays and immunohistochemical analysis.

**Results:**

According to the microarray profiles, PTP4A3/PRL-3 was upregulated in patients with poorly differentiated disease compared to patients with well-differentiated disease with hepatic backgrounds associated with hepatitis B or C. Validation analysis showed that the PTP4A3/PRL-3 mRNA and protein levels were significantly associated with poor differentiation (*P* < 0.0001), high serum α-fetoprotein (*P* < 0.01), high serum protein induced by vitamin K absence/antagonist-II (PIVKA-II), and hepatic vascular invasion (*P* < 0.05). The expression of PTP4A3/PRL-3 protein was also correlated with advanced cancer stages (*P* < 0.01); this resulted in a significantly poorer prognosis in both overall (*P* = 0.0024) and recurrence-free survival (*P* = 0.0227). According Cox regression univariate analysis, the positive expression of PTP4A3/PRL-3 was a poor risk prognostic factor (OS, *P* = 0.0031; recurrence-free survival, *P* = 0.0245). Cox regression multivariate analysis indicated that high PTP4A3/PRL-3 expression was an independent, unfavorable prognostic factor for overall survival (hazard ratio 0.542; *P* = 0.048).

**Conclusions:**

PTP4A3/PRL-3 might be closely associated with HCC progression, invasion, and metastasis. Its high expression had a negative impact on the prognosis of HCC patients. This strongly suggests that PTP4A3/PRL-3 should be considered as a prognostic factor. Further analysis should be pursued to evaluate it as a novel prognostic target.

**Electronic supplementary material:**

The online version of this article (doi:10.1245/s10434-012-2395-2) contains supplementary material, which is available to authorized users.

Hepatocellular carcinoma (HCC) is one of the most common malignant tumors worldwide.^1^ Although surgical resection is a potentially curative treatment for HCC, and despite improved diagnosis and advances in surgical and nonsurgical therapies, the clinical outcome of HCC remains poor.^2^^,^ 3 It is therefore crucial to understand the molecular mechanisms of this highly aggressive cancer, and finding molecular markers that can predict the tumor prognosis is important for developing new therapies.

Protein tyrosine phosphatase type IVA, member 3 (PTP4A3), is also known as phosphatase of regenerating liver-3 (PRL-3). It is a member of the PRL subgroup of protein tyrosine phosphatases, which are a large family of regulatory enzymes that function actively with protein tyrosine kinases in signaling pathways and participate in many fundamental physiological processes.^4^^–^ 6 Accumulating evidence suggests that PRL phosphatases, especially PRL-3, have been involved in the proliferation, growth regulation, increased cell motility, metastasis, and invasion in different types of cancers.^7^^–^ 9 It has been reported that PRL-3 was specifically overexpressed in liver metastases derived from colorectal cancer as well as invasive breast cancer, ovarian cancer, gastric cancer, esophageal squamous cell carcinoma, and squamous cell carcinoma of the cervix.^10^^–^ 13 Furthermore, PRL-3 was demonstrated to be a useful indicator for tumor recurrence and patient outcome in several human cancers.^14^^–^ 21 In HCC, PRL-3 was found to be overexpressed, and it was closely associated with tumor invasion and angiogenesis.^22^ However, up to now, the expression of PTP4A3/PRL-3 and its clinicopathological and prognostic significance in HCC have not been determined.

DNA microarray technology has enabled us to analyze the genome-wide profile of gene expression specific to malignant tumors. Such studies have the potential to lead to the development of a novel, molecular-targeted therapy for HCC.^23^ In the present study, PTP4A3/PRL-3 was obtained in poorly differentiated HCC tissues by examining the genome-wide expression profile of HCC generated by cDNA microarray. The results of training analyses, which were validated independently by TaqMan gene expression assays and immunohistochemically, and their clinicopathological significance, including the prognosis and recurrence of HCC, were investigated.

## Materials and Methods

### Patients and Preparation of Tissue Samples

We enrolled 256 patients undergoing partial hepatectomy for the first time of primary HCC between 2001 and 2010 at the Tokyo Medical and Dental University Hospital. The hepatitis virus status of these patients was as follows: 59 patients were positive for hepatitis B virus (HBV) surface antigen, 144 were positive for hepatitis C virus (HCV) antibody, and 53 were negative for both. In addition, 90 patients were pathologically diagnosed as having well-differentiated HCC, 91 moderately differentiated, and 75 poorly differentiated. In this study, we selected patients who were virus infected and whose HCC was either well or poorly differentiated, according to pathological diagnosis. A total of 142 primary liver tumor specimens (virus-negative patients and moderate-differentiation HCC tissues were excluded) and 10 normal liver specimens (which came from metastatic liver cancer patients whose origin tumor was diagnosed as colorectal cancer) were collected during surgery, snap frozen in liquid nitrogen, and stored at −80 °C. A portion of the tissue sample was fixed in 10 % formaldehyde solution and embedded in paraffin for histopathological analysis. Written informed consent was obtained from these patients, and the institutional review board approved the study. The patients were followed up with assays of their serum level of α-fetoprotein (AFP) and protein induced by vitamin K absence or antagonists II every month, and by ultrasonography, computed tomography (CT), and magnetic resonance imaging every 3 months.

### Microarray Gene Expression

Gene expression analysis was performed on the 42 primary HCC patients whose samples were used as training sets, and samples from the remaining 100 patients were used for validation analysis. Total RNA from those tissues was prepared with the RNeasy Mini Kit together with RNase-free DNase (Qiagen, Hiden, Germany). The integrity of the RNA obtained was assessed by an Agilent 2100 BioAnalyzer (Agilent Technologies, Palo Alto, CA, USA). All samples had a RNA integrity number (RIN) of >5.0. Using 2 μg of total RNA specimen tissues, cRNAs were prepared with a one-cycle target labeling and control reagents kit (Affymetrix, Santa Clara, CA, USA), and the microarray hybridization was carried out on Human Genome U133 2.0 chips (Affymetrix) according to the manufacturer’s protocol.

### Analysis of Gene Expression Data

A total of 42 training microarray data sets, including 15 HCC cases associated with HBV infection and 27 HCC cases associated with HCV infection, were normalized by the robust multiarray average (RMA) method by R statistical software, version 2.12.1, with the BioConductor package. Estimated gene expression levels were obtained in log2-transformed values, and 62 control probe sets were removed for further analysis. The fold change (FC) values were calculated by the ratio of the geometric means of the gene expression levels between the well-differentiated and poorly differentiated groups. A Wilcoxon rank sum test was performed to estimate the significance levels of the differences in gene expression between the two groups. A hierarchical clustering with the selected genes was performed with R software using the Euclidean distance and complete linkage method.

### Quantitative Real-Time Polymerase Chain Reaction (RT-PCR)

A total of 2 μg of tissue RNA was reverse transcribed to cDNA with a high-capacity cDNA reverse transcription kit (Applied Biosystems) according to the manufacturer’s instructions. The TaqMan MGB probe for PTP4A3/PRL-3 (Hs02341135_m1) was used for gene expression assays, and each PCR reaction was repeated at least three times. For the quantitative analysis of specific mRNA expression, CT values were calculated by 7500 SDS software. The data were analyzed by the ΔΔCT method (Applied Biosystems User Bulletin 2, 1997), and 18S rRNA was used as an internal control.

### Preparation of Cell Lines and RT-PCR of PTP4A3/PRL-3 mRNA

The human hepatoma cell lines SK-Hep1, Hep3-B, and PLC/PRF/5 were kindly provided by the American Type Culture Collection (Manassas, VA, USA). Huh1, Huh6, Huh7, HLE, HLF, HepG2, JHH1, JHH2, JHH4, and JHH5 were obtained from the Human Science Research Resources Bank (Osaka, Japan). The conditions of cell culture were reported previously.^24^ Total RNA was extracted with the RNeasy Mini Kit and reverse transcribed to cDNA with a high-capacity cDNA reverse transcription kit according to the manufacturer’s instructions.

### Immunohistochemical Staining

To further validate the expression of the candidate gene by microarray, immunohistochemical studies were performed. The primary antibody used was a polyclonal rabbit anti-human PTP4A3 (ab50276, Abcam, Cambridge, UK), diluted 1:400 in phosphate-buffered saline containing 1 % bovine serum albumin. The tissue sections were stained with an automated immunostainer (BenchMark XT; Ventana Medical Systems, Tucson, AZ, USA) by using heat-induced epitope retrieval and a standard diaminobenzidine detection kit (Ventana). The intensity of the cytoplasmic and nuclear membrane immunostaining for PTP4A3/PRL-3 was graded as follows: 0, no immunostaining; 1, immunostaining observed in <20 % of tumor cells; 2, moderate immunostaining observed in 20–49 %; and 3, strong and diffuse immunostaining observed in >50 % of tumor cells. A score of 0 or 1 was considered negative, whereas scores of 2 or 3 were considered positive. The immunohistochemical staining was evaluated under a light microscope by two independent investigators.

### Statistical Analysis

Statistical comparisons of the clinicopathological characteristics were performed by the χ^2^ test or Fisher’s exact test. Differences in mRNA levels between groups and the association between clinicopathological factors were analyzed by the Student *t* test. The data were expressed as mean ± standard deviation (SD). Fisher’s exact test was performed to estimate the associated between gene expression and the clinicopathological variables in each group. Overall survival (OS) and recurrence-free survival (RFS) curves were obtained by the Kaplan–Meier method and were compared with the log-rank test. Univariate and multivariate analyses were performed by Cox proportional hazard models.

## Results

### Strong Expression of PTP4A3/PRL-3 in of Poorly Differentiated Tumors was Detected by Gene Expression Profiling

**Fig. 1:**
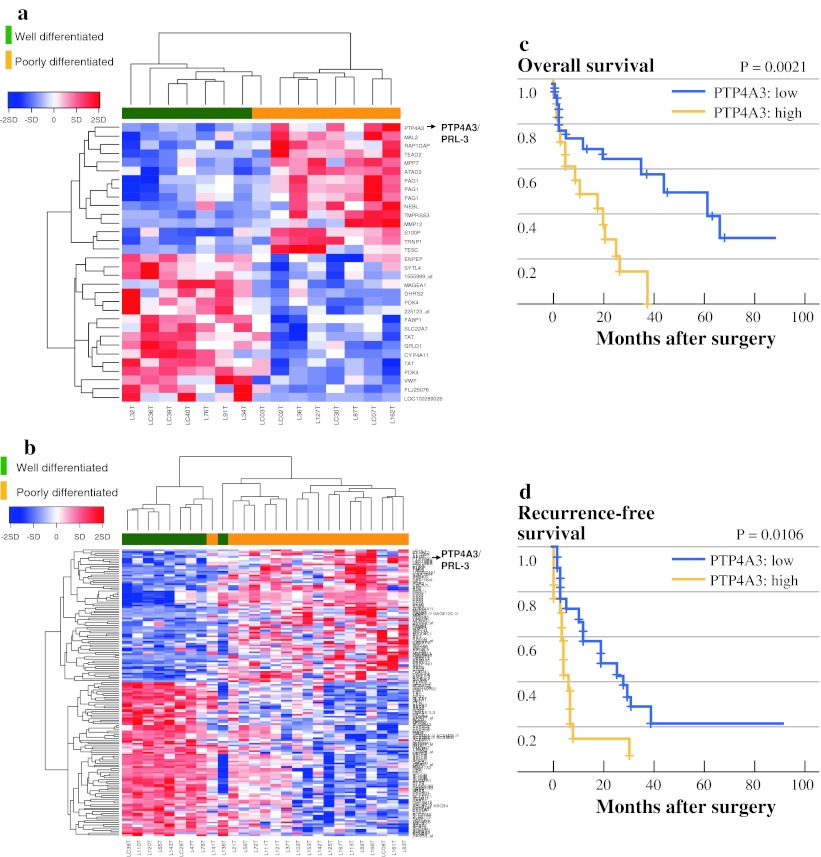
Microarray analysis of human hepatocellular carcinoma. **a** Hierarchical clustering of the gene expression for well-differentiated (*green*) and poorly differentiated (*orange*) HBV-associated HCC samples. **b** Hierarchical clustering of HCV-associated HCC samples. These genes were identified by the Wilcoxon signed rank test (*P* < 0.005), and a more than three-fold change between the two groups of well versus poorly differentiated HCCs. *Red* and *blue* represent relative overexpression and underexpression, respectively. **c**, **d** OS and RFS of 42 postoperative HCC patients associated with the expression of the PTP4A3/PRL-3 gene in cancerous liver tissue. The mean expression level for each gene was used as a cutoff value. *Solid* or *dotted* *lines* indicate the Kaplan–Meier curves for patients with relative overexpression and underexpression, respectively. The log-rank test was used to assess the statistical difference between the two groups (OS, *P* = 0.0021; RFS, *P* = 0.0106). *HBV* hepatitis B virus, *HCC* hepatocellular carcinoma, *HCV* hepatitis C virus, *OS* overall survival, *RFS* recurrence-free survival

Identification of those genes associated with the differentiation of the HCCs was performed by using the gene expression profiles obtained by the DNA microarray. First, the gene expression analysis was performed for 15 HCC cases associated with HBV (Fig. 1a) and 27 HCCs associated with HCV (Fig. 1b) according to a well or poor differentiation status of the tumor, respectively. In 54,613 probe sets, 32 probes (associated with HBV cases) (Supplementary Table S2) and 160 probes (associated with HCV cases) (Supplementary Table S3) that satisfied Wilcoxon *P* < 0.005 and FC > 3.0 were identified as differently expressed genes. As shown in Fig. 1a, b, the tumors were clearly divided by hierarchical clustering for well-differentiated (green) versus poorly differentiated (orange) cases. Next, we selected five probes (four genes) that had similar expression patterns associated with tumor differentiation, and the hepatic background was also associated HBV or HCV (*P* < 0.005, FC > 3.0) (Supplementary Table S4). Interestingly, the PTP4A3/PRL-3 gene was significantly upregulated in the poorly differentiated tumors in both HBV- and HCV-associated cases, indicating that PTP4A3/PRL-3 was the dominant molecule in the poorly differentiated HCC tumors. We then investigated the cumulative OS and tumor RFS curves of the 42 training cases for PTP4A3/PRL-3 expression after curative surgery. There were significant differences between the PTP4A3/PRL-3 high and low expression groups in terms of the OS (*P* = 0.0021) and RFS (*P* = 0.0106) rate, respectively (Fig. 1c, d).

### Clinicopathological and Molecular Factors Associated with HCC Survival and Recurrence

We examined the association between the clinicopathological factors and survivals of the 42 training patients with primary HCCs. The clinicopathological features of the 42 training patients according their differentiation status were studied by statistical analyses (Supplementary Table S1). The univariate Cox regression analysis demonstrated that the prothrombin time, AFP, portal vein invasion, advanced cancer stages, poor differentiation, and higher PTP4A3/PRL-3 expression correlated with both cumulative OS and RFS. The aspartate amino transferase (AST) and hepatic vein invasion correlated only with RFS. The other clinicopathological factors were not statistically significant (Table 1).

**Table 1:** Cox regression analysis of the overall and recurrence-free survival in the 42 training cases

	Overall survival		Recurrence-free survival	
	Univariate HR (95 % CI)	*P*	Univariate HR (95 % CI)	*P*
Clinicopathological factors				
Age (years, mean ± SD)	0.978 (0.942–1.015)	0.2392	0.990 (0.951–1.031)	0.6367
Gender (female vs. male)	0.830 (0.331–2.212)	0.7089	0.907 (0.387–2.124)	0.822
Virus infection (HBV vs. HCV)	1.331 (0.606–2.923)	0.4768	0.940 (0.438–2.016)	0.8738
AST (IU/l, mean ± SD)	1.005 (0.994–1.016)	0.3639	1.011 (1.000–1.022)	0.0423
ALT (IU/l, mean ± SD)	1.003 (0990–1.016)	0.6777	1.006 (0.993–1.019)	0.3804
Plt (×10^9^/l, mean ± SD)	1.026 (0.956–1.100)	0.4811	1.025 (0.959–1.094)	0.468
ICG-R15 (%, mean ± SD)	1.006 (0.976–1.037)	0.6863	1.016 (0.986–1.046)	0.3063
PT (%, mean ± SD)	0.945 (0.913–0.978)	0.0013	0.967 (0.937–0.998)	0.0396
T. bil (mg/dl, mean ± SD)	1.942 (1.165–3.237)	0.0109	1.144 (0.649–2.018)	0.6411
Alb (g/dl, mean ± SD)	0.624 (0.289–1.348)	0.2302	0.690 (0.326–1.458)	0.3311
AFP (ng/ml, log10)	1.769 (1.312–2.385)	0.0002	1.767 (1.338–2.332)	<0.0001
PIVKA-II (mAU/ml, log10)	1.196 (0.827–1.730)	0.3411	1.282 (0.891–1.846)	0.1813
Tumor max size (cm, mean ± SD)	1.092 (0.957–1.246)	0.1900	1.105 (0.982–1.243)	0.0965
Multiple versus solitary	0.936 (0.376–2.332)	0.8870	1.305 (0.577–2.954)	0.5227
Capsular formation (−) versus (+)	1.632 (0.761–3.501)	0.2083	0.902 (0.413–1.974)	0.7968
Capsular invasion (−) versus (+)	1.752 (0.814–3.772)	0.1517	1.295 (0.615–2.726)	0.4967
Portal vein invasion (pvp)	0.307 (0.138–0.683)	0.0038	0.185 (0.084–0.405)	<0.0001
Hepatic vein invasion (pvv)	0.663 (0.244–1.806)	0.4212	0.374 (0.156–0.897)	0.0276
Vascular invasion (pvp/pvv)	0.331 (0.114–0.777)	0.0112	0.179 (0.080–0.400)	<0.0001
Stage (III + IV) versus (I + II)	0.331 (0.114–0.777)	0.0112	0.344 (0.160–0.740)	0.0063
Degree of differentiation (poor vs. well)	4.146 (1.526–11.263)	0.0053	3.678 (1.596–8.477)	0.0022
Molecule factor				
PTP4A3/PRL-3 expression (high)	3.563 (1.5.09–8.411)	0.0037	2.598 (1.217–5.545)	0.0136

*HR* hazard ratio, *CI* confidence interval, *HBV* hepatitis B virus, *HCV* hepatitis C virus, *AST* aspartate amino transferase, *ALT* alanine aminotransferase, *PLT* platelet, *ICG*-*R15* indocyanine green retention rate at 15 min, *PT* prothrombin time, *T. bil* total bilirubin, *Alb* albumin, *AFP* α-fetoprotein, *PIVKA*-*II* protein induced by vitamin K absence or antagonists II

### Validation Study by TaqMan Gene Expression Assays

**Fig. 2:**
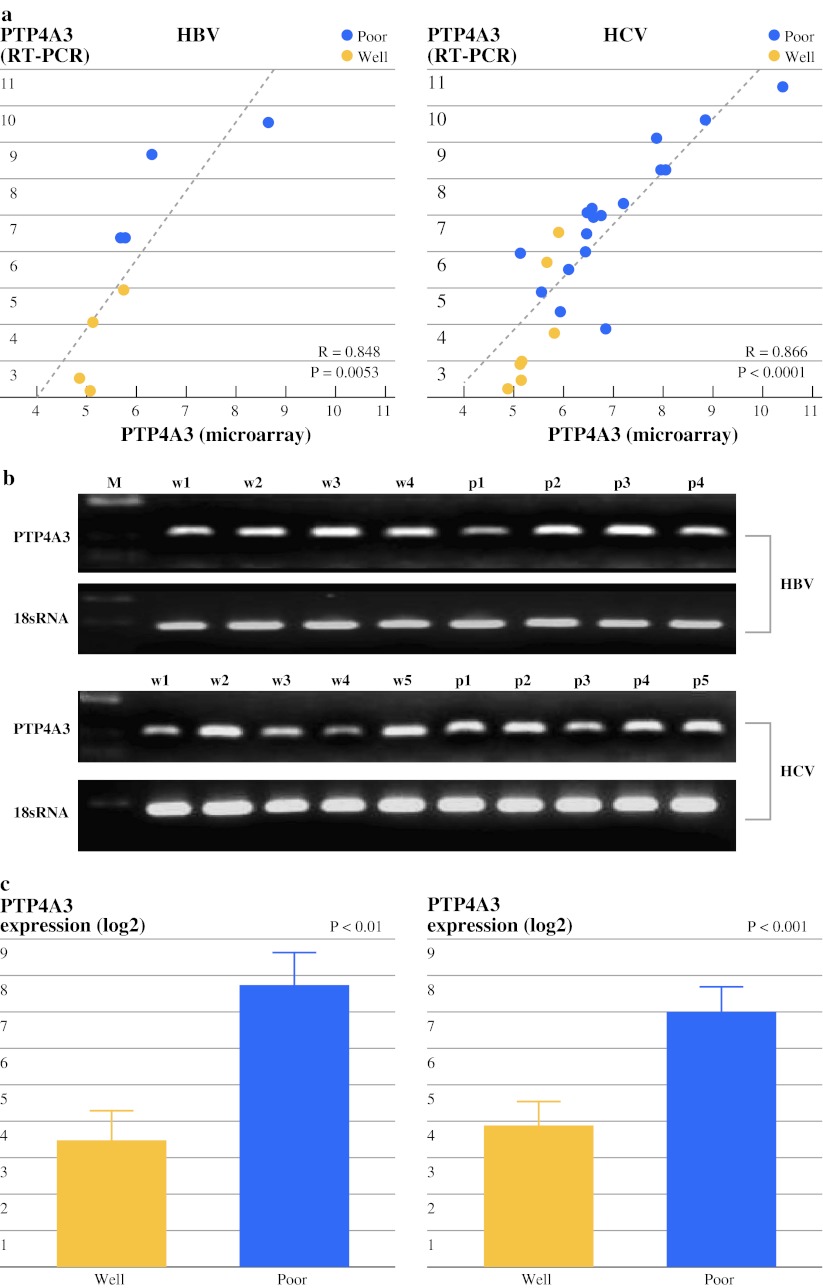

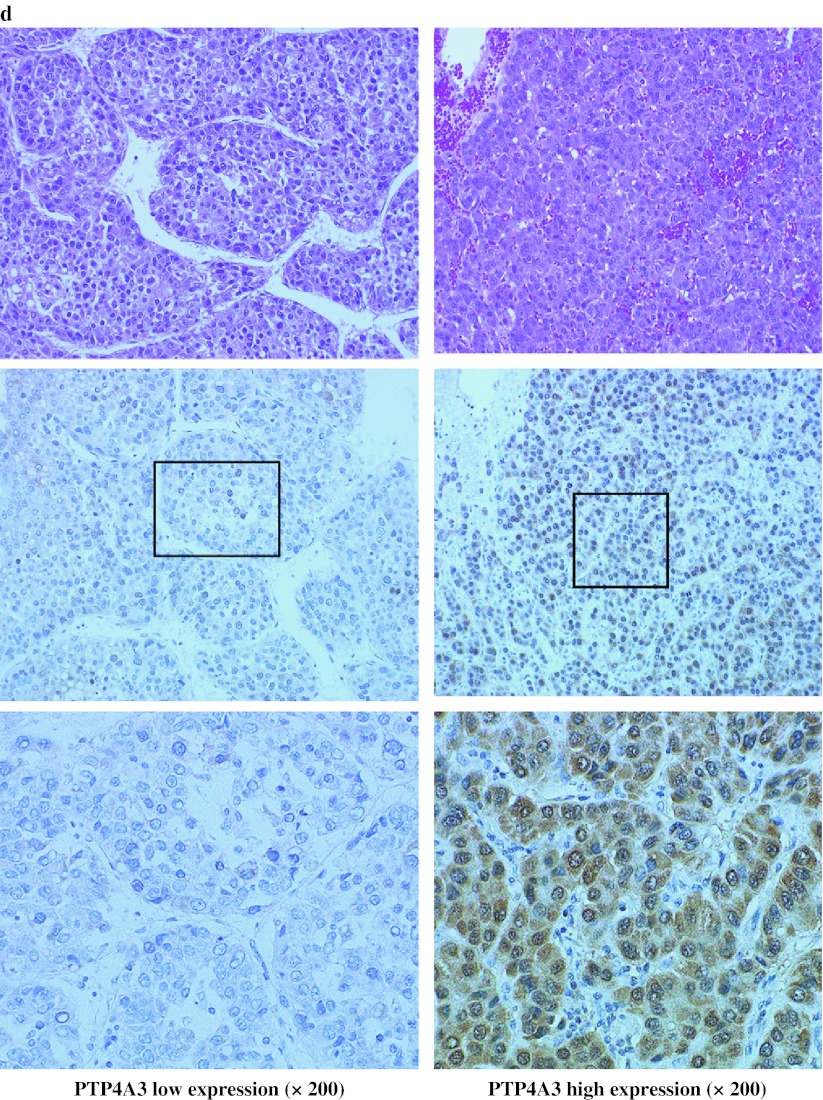
Training study: expression of PTP4A3/PRL-3 in HCC. **a** Correlation of the test samples for PTP4A3/PRL-3 mRNA expression by cDNA microarray and TaqMan gene expression assays (*left* HBV-associated patients, *right* HCV-associated patients). **b** RT-PCR products were detected by agarose gel electrophoreses stratified by HBV- or HCV-associated HCC (*w* Well-differentiated, *p* poorly differentiated). **c** The mRNA expression levels of PTP4A3/PRL-3 were compared between poorly differentiated and well-differentiated disease according to the presence of HBV (*left*) associated (*P* < 0.01) or HCV (*right*) associated (*P* < 0.001) HCC, respectively. **d** Immunohistochemical analysis of PTP4A3/PRL-3 in HCC tissues from the test samples. *Top* HE staining. *Middle*, *bottom* PTP4A3/PRL-3-high (*right*) or low (*left*) expression (original magnification, ×100, ×200)

**Fig. 3:**
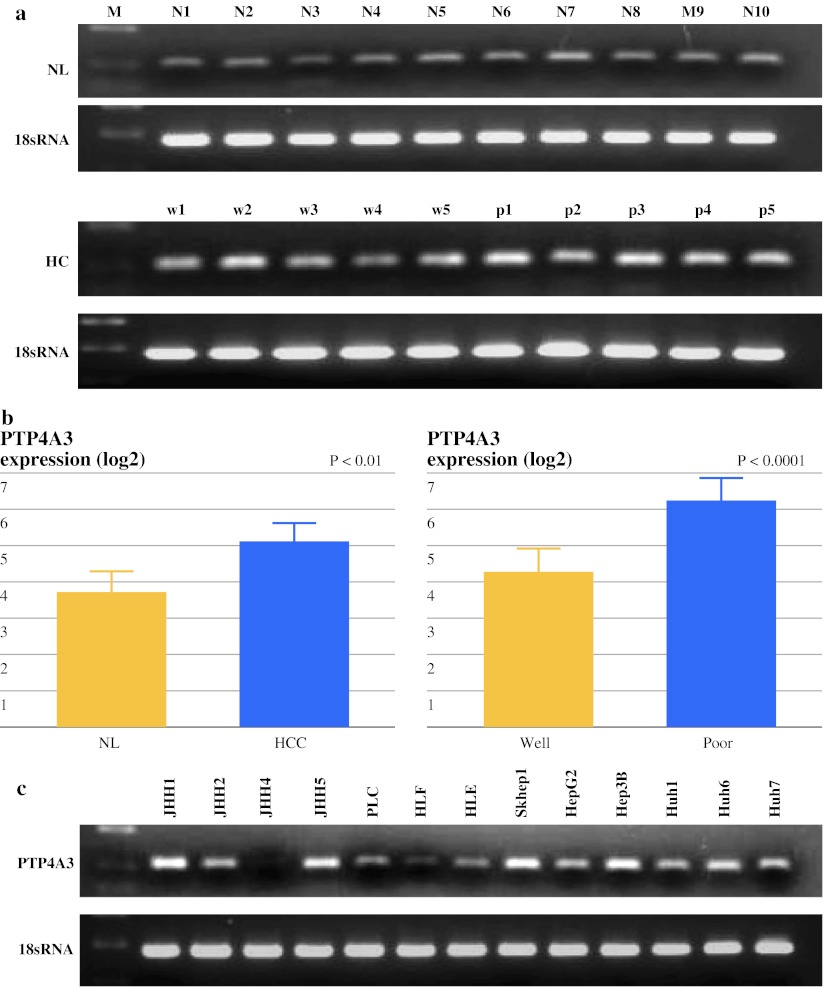

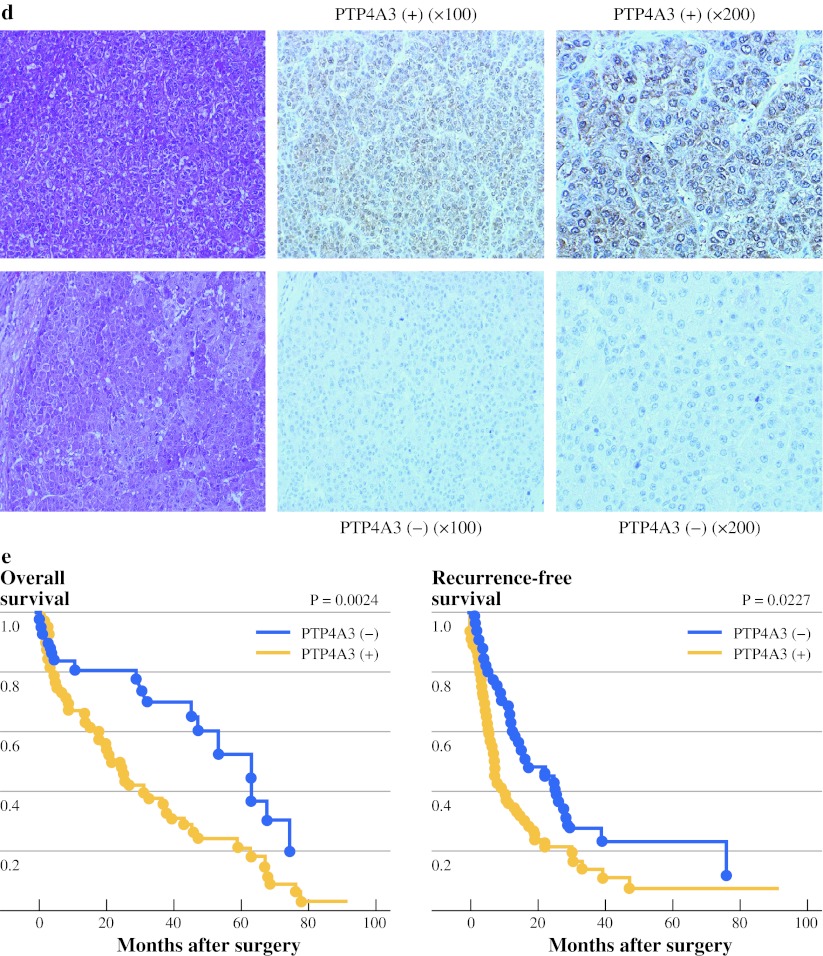
Validation study: expression of PTP4A3/PRL-3 in human HCC. **a** RT-PCR products were detected by agarose gel electrophoreses stratified by normal liver and HCC (*w* well, *p* poor) tissues. **b** The mRNA expression levels of PTP4A3/PRL-3 were compared between the HCC and normal liver tissues (NL) (*P* < 0.01), and between well-differentiated and poorly differentiated HCC tissues (*P* < 0.0001) by TaqMan gene expression assay. **c** The expression of PTP4A3/PRL-3 in hepatoma cell lines. **d** Immunohistochemical analysis of PTP4A3/PRL-3 in HCC tissues from the validation samples. A representative positive immunostaining case (*top*) and a negative immunostaining case (*bottom*) are shown. **e** Kaplan–Meier estimates for the OS and RFS times with respect to PTP4A3/PRL-3 protein expression in primary HCCs. The survival rate of patients with positive or negative PTP4A3/PRL-3 expression are indicated by *solid* or *dotted lines*, respectively. The log-rank test results revealed a significant difference in OS rates between the two groups (*P* = 0.0024), and also a significant difference was observed for the RFS rates between the two groups (*P* = 0.0227). *HCC* hepatocellular carcinoma, *OS* overall survival, *RFS* recurrence-free survival

According to the training microarray analysis, the PTP4A3/PRL-3 gene was closely correlated with poor differentiation, poorer OS, and recurrence of HCC. To confirm the microarray findings, the results were independently validated by TaqMan gene expression assays. We also tested the microarray results in training sets. There was a significant correlation between the cDNA microarray and TaqMan gene expression assays results in terms of PTP4A3/PRL-3 expression (Fig. 2a). The expression of PTP4A3/PRL-3 in poorly differentiated HCC tissues was significantly higher than in well-differentiated tissues of HCC patients associated with HBV (*P* < 0.01) or HCV (*P* < 0.001) (Fig. 2c). This result was validated independently in HCC tumor tissues from 83 patients and in 10 normal liver specimens. The mRNA expression levels of PTP4A3/PRL-3 were statistically significantly different between HCC and normal liver specimens (5.29 ± 1.43 vs. 3.86 ± 0.76, *P* = 0.0035, Fig. 3b, left), PTP4A3/PRL-3 was more highly expressed in poorly differentiated disease than in well-differentiated disease (6.21 ± 2.25 vs. 4.17 ± 1.70, *P* < 0.0001, Fig. 3b, right). The clinicopathological significance of PTP4A3/PRL-3 mRNA expression was also analyzed. Depending on the expression level (means ± SD) of PTP4A3/PRL-3 mRNA, the clinicopathological factors were divided into two groups. PTP4A3/PRL-3 mRNA was significantly correlated with virus infection (*P* = 0.0422), serum AFP (*P* = 0.0047), PIVKA-II (*P* = 0.0259), portal vein invasion (*P* = 0.0156), and vascular invasion (*P* = 0.0192) (Table 2). There were no statistically significant differences between age, gender, size of tumor, number of tumors, tumor infiltration into the capsule, and stages of the tumor.

**Table 2:** Association between PTP4A3/PRL-3 expression and clinicopathological factors in HCC patients

Variable	PTP4A3 mRNA			PTP4A3 protein			
	*n*	Mean ± SD	*P*	*n*	Negative	Positive	*P*
Age (years)							
<60	20	5.10 ± 2.23	0.9009	28	11	17	0.6609
≥60	63	5.17 ± 2.23		72	32	40	
Gender							
Male	60	5.32 ± 2.38	0.2895	72	25	47	0.0126
Female	23	4.74 ± 1.70		28	18	10	
Virus infection							
HBV	25	4.40 ± 2.46	0.0422	33	18	15	0.1333
HCV	58	5.48 ± 2.05		67	25	42	
No. of tumors							
Solitary	53	5.13 ± 2.10	0.8942	71	33	38	0.3736
Multiple	30	5.20 ± 2.47		29	10	19	
Tumor size (cm)							
<5.0	58	4.86 ± 1.92	0.0706	70	33	37	0.2710
≥5.0	25	5.83 ± 2.73		30	10	20	
AFP (ng/ml)							
<20	34	4.22 ± 1.85	0.0011	40	26	14	0.0004
≥20	49	5.80 ± 2.24		60	17	43	
<100	55	4.67 ± 2.07	0.0047	65	34	31	0.0118
≥100	28	6.11 ± 2.23		35	9	26	
PIVKA-II (mAU/ml)							
<100	39	4.58 ± 2.38	0.0259	46	23	23	0.2268
≥100	44	5.66 ± 1.96		54	20	34	
<1000	59	4.82 ± 2.16	0.0313	74	37	37	0.0214
≥1000	24	5.97 ± 2.20		26	6	20	
Differentiation							
Well	43	4.17 ± 1.70	<0.0001	46	30	16	<0.0001
Poor	40	6.21 ± 2.25		54	13	41	
Infiltration to capsule (fc-inf)							
Absent	35	5.30 ± 2.49	0.6177	48	20	28	0.8416
Present	48	5.05 ± 2.02		52	23	29	
Portal vein invasion (pvp)							
Absent	60	4.79 ± 2.21	0.0156	72	35	37	0.0770
Present	23	6.10 ± 2.00		28	8	20	
Hepatic vein invasion (pvv)							
Absent	71	5.00 ± 2.28	0.1310	87	41	46	0.0374
Present	12	6.05 ± 1.65		13	2	11	
Vascular invasion (pvp/pvv)							
Absent	53	4.73 ± 2.28	0.0192	66	34	32	0.0198
Present	30	5.91 ± 1.93		34	9	25	
Tumor stages							
I + II	33	4.75 ± 1.83	0.1795	44	26	18	0.0047
III + IV	50	5.42 ± 2.43		56	17	39	

*HBV* hepatitis B virus, *HCV* hepatitis C virus, *AFP* α-fetoprotein, *PIVKA*-*II* protein induced by vitamin K absence or antagonists II

### Expression of PTP4A3/PRL-3 in HCC Cell Lines

The expression level of PTP4A3/PRL-3 mRNA was evaluated in 13 hepatoma cell lines. The PTP4A3/PRL-3 mRNA was highly expressed in HCC cell lines, and there were different expression patterns (Fig. 3c). In 2 of 13 cells, the PTP4A3/PRL-3 mRNA levels were lower than in the known cell lines such as HLE and HLF derived from epithelial and fibroblastic colonies in cultures of the same undifferentiated hepatomas and did not produce AFP and albumin. The other cells were derived from differentiated hepatomas and produced AFP.

### Immunohistochemical Detection of PTP4A3/PRL-3 in HCC Tissues and Association with Clinicopathological Variables

Next, an immunohistochemical analysis was performed for the evaluation of the clinical significance of PTP4A3/PRL-3 expression by using tissue samples from 100 patients with HCC. We also tested the immunoreactivity of PTP4A3/PRL-3 in the same microarray test samples. The results for PTP4A3/PRL-3 immunostaining are shown in Fig. 2d. PTP4A3/PRL-3 protein was mainly localized in the cytoplasm and nuclear membrane of the cancer cells, and rarely nuclear staining was also seen. Consequently, the specific overexpression of PTP4A3/PRL-3 was recognized in 57 of 100 cases (Fig. 3d).

The clinicopathological significance of PTP4A3/PRL-3 expression was then evaluated in the PTP4A3/PRL-3-positive group (*n* = 57) and compared to the negative group (*n* = 43) of HCC patients (Table 2). The protein expression of PTP4A3/PRL-3 was significantly correlated with tumor differentiation (*P* < 0.0001), serum AFP (*P* = 0.0118), high serum PIVKA-II (*P* = 0.0214), hepatic vein invasion (*P* = 0.0374), tumor vascular invasion (*P* = 0.0198), and advanced cancer stages (*P* = 0.0047).

### Overexpression of PTP4A3 Protein in HCC Tissues Associated with a Poor Prognosis

To examine the prognostic significance of PTP4A3/PRL-3 expression, we performed a validation study on 100 patients with HCC. According to the immunostaining analysis of HCC tissues, the OS and RFS survival were then compared between the two groups. The PTP4A3/PRL-3-positive group demonstrated a significantly poorer survival than the PTP4A3/PRL-3-negative group for both OS (*P* = 0.0024) and RFS (*P* = 0.0227). We also investigated the association between the OS and RFS rates and clinicopathological factors, and we performed univariate and multivariate Cox regression analyses (Table 3). The OS was statistically significantly correlated with seven factors such as AST, serum AFP, tumor size, tumor differentiation, portal vein invasion, stage of tumor, and PTP4A3/PRL-3 expression. On the multivariate analysis, PTP4A3/PRL-3 expression (hazard ratio [HR] 0.542; *P* = 0.048), portal vein invasion (HR 0.470; *P* = 0.023), and AST (HR 1.006; *P* = 0.048) were statistically significant prognostic factors for OS, but not for the RFS rate (Table 3).

**Table 3:** Cox regression analysis of overall and recurrence-free survival in 100 validation cases

	Overall survival				Recurrence-free survival			
	Univariate analysis		Variable selection		Univariate analysis		Variable selection	
	HR (95 % CI)	*P*	HR (95 % CI)	*P*	HR (95 % CI)	*P*	HR (95 % CI)	*P*
Clinicopathological factors								
Age (years, mean ± SD)	0.996 (0.968–1.025)	0.7901			0.993 (0.966–1.021)	0.6375		
Gender (female/male)	0.712 (0.412–1.229)	0.2226			0.755 (0.456–1.250)	0.2752		
Virus infection (HBV vs. HCV)	1.142 (0.673–1.938)	0.6231			1.187 (0.730–1.931)	0.4892		
AST (IU/l, mean ± SD)	1.007 (1.001–1.014)	0.0192	1.006 (1.000–1.013)	0.048	1.005 (0.998–1.012)	0.1327		
ALT (IU/l, mean ± SD)	1.005 (0.999–1.011)	0.1096			1.004 (0.997–1.010)	0.2977		
Plt (×10^9^/l, mean ± SD)	1.017 (0.987–1.047)	0.2822			0.999 (0.970–1.030)	0.9601		
ICG-R15 (%, mean ± SD)	0.997 (0.979–1.015)	0.7119			0.998 (0.982–1.015)	0.8202		
PT (%, mean ± SD)	0.991 (0.978–1.005)	0.2113			0.996 (0.984–1.008)	0.4896		
T. bil (mg/dl, mean ± SD)	1.212 (0.859–1.709)	0.2741			0.964 (0.676–1.369)	0.838		
Alb (g/dl, mean ± SD)	1.062 (0.756–1.491)	0.7287			0.973 (0.732–1.293)	0.8495		
AFP, ng/ml (≥20 vs. <20)	0.523 (0.309–0.885)	0.0157	0.939 (0.526–1.676)	0.832	0.505 (0.308–0.827)	0.0067	0.815 (0.453–1.466)	0.4946
PIVKA-II, mAU/ml (≥40 vs.<40)	0.842 (0.509–1.391)	0.5009			1.091 (0.681–1.749)	0.7161		
Maximum tumor size (cm, mean ± SD)	1.149 (1.055–1.252)	0.0014	1.064 (0.956–1.185)	0.2544	1.111 (1.023–1.207)	0.0127	0.952 (0.847–1.071)	0.4127
No. of tumors (multiple vs. solitary)	0.836 (0.483–1.515)	0.5930			1.312 (0.796–2.165)	0.2869		
Differentiation (poor vs. well)	2.364 (1.412–3.968)	0.0011	1.266 (0.661–2.424)	0.477	1.931 (1.206–3.094)	0.0066	1.026 (0.562–1.872)	0.9345
Capsular invasion (pfc-inf) (∓)	1.344 (0.822–2.197)	0.2388			1.192 (0.750–1.893)	0.4583		
Portal vein invasion	0.304 (0.177–0.523)	<0.0001	0.470 (0.245–0.901)	0.023	0.251 (0.150–0.420)	<0.0001	0.316 (0.165–0.603)	0.0005
Hepatic vein invasion	0.823 (0.403–1.682)	0.5933			0.472 (0.253–0.879)	0.0181	0.654 (0.326–1.312)	0.2321
Stage (III + IV) versus (I + II)	0.400 (0.239–0.658)	0.0005	0.661 (0.352–1.239)	0.196	0.410 (0.253–0.664)	0.0003	0.489 (0.269–0.890)	0.0191
Molecular factor								
PTP4A3/PRL-3 protein expression	0.440 (0.255–0.759)	0.0031	0.542 (0.294–0.997)	0.048	0.578 (0.359–0.932)	0.0245	1.000 (0.556–1.798)	0.9994

*HR* hazard ratio, *CI* confidence interval, *HBV* hepatitis B virus, *HCV* hepatitis C virus, *AST* aspartate amino transferase, *ALT* alanine aminotransferase, *PLT* platelet, *PT* prothrombin time, *ICG*-*R15* indocyanine green retention rate at 15 min, *T. bil* total bilirubin, *Alb* albumin, *AFP* α-fetoprotein, *PIVKA*-*II* protein induced by vitamin K absence or antagonists II

## Discussion

In the present study, the gene expression profiles of PTP4A3/PRL-3 were obtained in poorly differentiated and well-differentiated HCC, regardless of the background of the virus. On the basis of the training analysis, strong PTP4A3/PRL-3 expression was significantly correlated with the cumulative survival and recurrence-free rates of the patients (Fig. 1c, d; Table 1). This result was validated independently by TaqMan gene expression assays and immunostaining. We also studied the significance of PTP4A3/PRL-3 mRNA and protein expression, from a clinical viewpoint, for the progression of HCC. The overexpression of PTP4A3/PRL-3 was significantly correlated with the serum levels of AFP and PIVKA-II, tumor vascular invasion, and advanced cancer stages in HCC patients. Similar results have been reported by Zhao et al.,^22^ suggesting that PRL-3 expression was correlated with angiogenesis and invasion through an upregulation of matrix metalloproteinases (MMPs) or a downregulation of E-cadherin by using limited HCC cases. PRL-3 expression has also been reported in colon, breast, ovarian, and gastric cancers, where its presence seems to have an important role in the acquisition of metastatic potential.^11^^,^ 15 ^–^ 21 In our studies, the clinical significance of PTP4A3/PRL-3 expression was noted and correlated significantly with poor tumor differentiation (*P* < 0.0001) and the OS and RFS of HCC patients (Fig. 3e). PTP4A3/PRL-3 expression also showed a significant correlation with a poor clinical outcome (Table 3).

PTP4A3/PRL-3 plays multiple roles in cancer metastasis. By activating a series of intracellular signaling pathways, PRL-3 induces cell differentiation, proliferation, invasion, and metastasis.^7^^–^ 9 Invasion through the basement membrane and interstitial extracellular matrix is another key event for metastatic progression, which requires the action of a series of proteolytic enzymes, the MMPs. The MMP family is closely correlated with metastatic potential; they can degrade denatured collagens and type IV collagen present in the basement membrane.^25^ In our training microarray studies (Supplementary Table S2), MMP12 was also highly expressed in poorly differentiated patients with HCC. In addition, other members of the metalloproteinase family such as MMP1, MMP9, and MMP10 (Wilcoxon *P* < 0.05; FC > 1.5) were also upregulated in poorly differentiated patients (data not shown in Supplementary Table S2). We found significantly positive correlations between PTP4A3/PRL-3 and MMP12 (*r* = 0.721, *P* < 0.0001), MMP1 (*r* = 0.514, *P* = 0.0004), MMP9 (*r* = 0.501, *P* = 0.0006), and MMP10 (*r* = 0.347, *P* = 0.0236) (Supplementary Fig. S1). Several studies have suggested that a downregulation of E-cadherin-mediated intercellular adhesion increased tumor differentiation, invasion, and metastasis and led to a poor prognosis in human cancers, including HCC.^26^^,^ 27 At present, our microarray studies also showed that the downregulation of E-cadherin in tumor tissues was associated with hepatic vascular invasion, moderate to poor differentiation, and the recurrence of patients with HCC (data not shown). However, there was no statistical correlation between PTP4A3/PRL-3 expression and E-cadherin expression on the basis of the training sets analysis (Supplementary Fig. S1). This result suggested a role for PTP4A3/PRL-3 overexpression in HCC, and its ectopic expression in different cell types is correlated with the induction of metastatic phenotypes, such as invasion and motility capabilities. PTP4A3/PRL-3 expression was predominantly correlated with poorly differentiated tumor cells and increased invasion potential through an upregulation of MMPs.

In the present study, we showed that PTP4A3/PRL-3 was predominantly localized in the cytoplasm and nuclear membrane by immunostaining. The expression of membrane-associated PRL-3 may induce a dephosphorylation of target substrates at the cell membrane, thus modulating the organization of the plasma membrane in such a way to promote cell motility and invasion.^8^ The membrane structures associated with PRL-3 including ruffles, protrusions, and some vacuolar-like membrane extensions could represent another opportunity for intervention. These membrane structures have been demonstrated to play a role in cell movement and invasion.^28^

Several recent studies have demonstrated the capability of PRL-3 to regulate multiple signaling cascades, including the PI3K-Akt, Rho, and Csk/Src pathways, which are crucial for both cell cycle progression and cell migration.^29^^,^ 30 PRL-3-enhanced cell migration is dependent on the preservation of its catalytic function, and the consensus phosphatase motif will potentially be a therapeutic target. Prenylation-dependent association could effectively block the function of PRL-3 in cell migration and invasion.^8^ Aberrant PTP4A3/PRL-3 expression may be closely related to poor tumor differentiation, tumor invasion, and poorer survival in HCC patients, and this accelerated hepatocarcinogenesis may be positively regulated by hepatoepithelial cell migration, thus promoting tumor progression and the establishment of the new vasculature needed for tumor survival and expansion. However, at this time, we are not able to analyze any data to support this hypothesis. Further study is required to verify these possibilities.

In conclusion, PTP4A3/PRL-3 was identified as one of the overexpressed molecules in HCC tissues. PTP4A3/PRL-3 might be closely associated with HCC progression, invasion, and metastasis, and its strong expression had a negative impact on the prognosis of HCC patients. This suggests that PRL-3 should be considered as a prognostic factor. Further attention should be paid to abnormalities in phosphatase of PRL-3 in HCC progression as a potential target for therapy.

## Electronic supplementary material

Below is the link to the electronic supplementary material.

Supplementary material 1 (PDF 161 kb)
