# Supplementary material for: Upregulation of Protein Tyrosine Phosphatase Type IVA Member 3 (PTP4A3/PRL-3) is Associated with Tumor Differentiation and a Poor Prognosis in Human Hepatocellular Carcinoma
Source: Ann Surg Oncol. 2012 Oct 13;20(1):305–17. doi: 10.1245/s10434-012-2395-2 (PMC3528959; doi:10.1245/s10434-012-2395-2)
Supplement: Supplementary file 1 — Supplementary material 1 (PDF 161 kb) [file 10434_2012_2395_MOESM1_ESM.pdf]

TABLE-S1. Clinicopathological features of the 42 training cases according to the degree of differentiation of the tumor

Clinicopathologic factors	Well (n=16)	Poor (n=26)	*P* value
Age (y, mean±SD)	66.4±6.38	60.8±12.0	0.0951
Gender (male/female)	11/5	23/3	0.2233
Viral infection (HBV/HCV)	7/9	8/18	0.5113
AST (IU/L, mean±SD)	51.0±23.8	58.9±33.6	0.4151
ALT (IU/L, mean±SD)	47.8±25.1	44.5±33.6	0.7387
Plt (x109/L, mean±SD)	11.2±4.51	15.2±6.39	0.0336
ICG-R15 (%, mean±SD)	17.2±6.99	20.0±14.5	0.4731
PT% (mean±SD)	88.0±14.1	79.4±11.3	0.0334
T.bil (mg/dl, mean±SD)	0.74±0.20	1.10±1.43	0.3244
Alb (g/dl, mean±SD)	3.94±0.43	3.91±0.51	0.8337
AFP (ng/ml, log10)	1.08±0.58	2.49±1.37	0.0003
PIVKA-II (mAU/ml, log10)	1.94±0.90	2.67±1.22	0.0444
Tumor max size (cm, mean±SD)	3.83±2.27	5.03±3.26	0.2028
Tumor max size (cm, ≧2.0vs <2.0)	16/0	24/2	0.5168
Tumor max size (cm, ≧5.0vs <5.0)	1/15	11/15	0.0150
Solitary or Multiple	12/4	19/7	1.000
Capsular formation (pfc) (-/+)	6/10	9/17	1.000
Capsular invasion (pfc-inf) (-/+)	7/9	11/15	1.000
Portal vein invasion (pvp) (-/+)	15/1	13/13	0.0058
Hepatic vein invasion (pvv) (-/+)	14/2	21/5	0.6897
Vascular invasion (pvp/pvv) (-/+)	14/2	10/16	0.0032
Stages (I+II/III+IV)	10/6	7/19	0.0291
Recurrence (absent/present)	7/9	5/21	0.1581

NOTE: AST, aspartate amino transferase; ALT, alanine aminotransferase; PLT, plat
ICG-R15, Indocyanine Green Retention Rate at 15 min; PT%, Prothrombin Time;
T.bil, Total bilirubin; Alb, albumin; AFP, α-fetoprotein; PIVKAII, protein induced b
vitamin K absence or antagonists II; positive, +; negative, -;

TABLE-S2. Thirty-two probe sets identified as differently expressed genes that satisfied p <0.005, FC >3.0 by the Wilcoxon rank sum test between poorly and well differentiated HBV associated patients with HCC

Probe set	Symbol	Title	p-value	Fold-change
225123_at	---	---	0.0003	0.30
225207_at	PDK4	pyruvate dehydrogenase kinase, isozyme 4	0.0003	0.16
237737_at	LOC10028902(	similar to hCG1744891	0.0003	0.18
205960_at	PDK4	pyruvate dehydrogenase kinase, isozyme 4	0.0006	0.23
1555989_at	---	---	0.0012	0.33
203911_at	RAP1GAP	RAP1 GTPase activating protein	0.0012	4.96
204351_at	S100P	S100 calcium binding protein P	0.0012	22.46
206574_s_at	PTP4A3	protein tyrosine phosphatase type IVA, member 3	0.0012	3.14
225626_at	PAG1	phosphoprotein associated with glycosphingolipid microdomains 1	0.0012	6.47
227354_at	PAG1	phosphoprotein associated with glycosphingolipid microdomains 1	0.0012	5.59
1554931_at	CYP4A11	cytochrome P450, family 4, subfamily A, polypeptide	0.0022	0.30
202112_at	VWF	von Willebrand factor	0.0022	0.25
206916_x_at	TAT	tyrosine aminotransferase	0.0022	0.27
220177_s_at	TMPRSS3	transmembrane protease, serine 3	0.0022	4.18
221661_at	SLC22A7	solute carrier family 22 (organic anion transporter), member 7	0.0022	0.24
226612_at	FLJ25076	probable ubiquitin-conjugating enzyme E2 FLJ25076	0.0022	0.29
231693_at	FABP1	Fatty acid binding protein 1, liver	0.0022	0.30
203961_at	NEBL	nebulette	0.0037	6.62
204580_at	MMP12	matrix metallopeptidase 12 (macrophage elastase)	0.0037	3.21
204845_s_at	ENPEP	glutamyl aminopeptidase (aminopeptidase A)	0.0037	0.28
206265_s_at	GPLD1	glycosylphosphatidylinositol specific phospholipase I	0.0037	0.18
207325_x_at	MAGEA1	melanoma antigen family A, 1 (directs expression of antigen MZ2-E)	0.0037	0.22
214079_at	DHRS2	dehydrogenase/reductase (SDR family) member 2	0.0037	0.09
214413_at	TAT	Tyrosine aminotransferase	0.0037	0.16
218872_at	TESC	tescalcin	0.0037	5.09
224650_at	MAL2	mal, T-cell differentiation protein 2	0.0037	3.33
225622_at	PAG1	phosphoprotein associated with glycosphingolipid microdomains 1	0.0037	3.75
226408_at	TEAD2	TEA domain family member 2	0.0037	3.19
227703_s_at	SYTL4	synaptotagmin-like 4	0.0037	0.27
227862_at	TRNP1	TMF1-regulated nuclear protein 1	0.0037	7.40
228401_at	ATAD2	ATPase family, AAA domain containing 2	0.0037	3.19
238778_at	MPP7	membrane protein, palmitoylated 7 (MAGUK p55 subfamily member 7)	0.0037	3.95

NOTE: HBV, hepatitis B virus

TABLE-S3. One hundred-sixty probe sets identified as differently expressed genes that satisfied p <0.005, FC >3.0 by the Wilcoxon rank sum test between poorly and well differentiated HCV associated patients with HCC.

Probe set	Symbol	Title	p-value	Fold-change
219232_s_at	EGLN3	egl nine homolog 3 (C. elegans)	<0.0001	3.28
204288_s_at	SORBS2	sorbin and SH3 domain containing 2	<0.0001	0.32
204351_at	S100P	S100 calcium binding protein P	<0.0001	32.93
219863_at	HERC5	hect domain and RLD 5	<0.0001	0.32
225728_at	SORBS2	sorbin and SH3 domain containing 2	<0.0001	0.29
206574_s_at	PTP4A3	protein tyrosine phosphatase type IVA, member 3	<0.0001	3.42
205047_s_at	ASNS	asparagine synthetase	<0.0001	7.86
214719_at	SLC46A3	solute carrier family 46, member 3	<0.0001	0.20
227803_at	ENPP5	ectonucleotide pyrophosphatase/phosphodiesterase 5 (putative f	<0.0001	4.37
228293_at	DEPDC7	DEP domain containing 7	<0.0001	0.24
240253_at	---	---	<0.0001	0.28
205768_s_at	SLC27A2	solute carrier family 27 (fatty acid transporter), member 2	<0.0001	0.19
228912_at	VIL1	Villin 1	<0.0001	5.31
205769_at	SLC27A2	solute carrier family 27 (fatty acid transporter), member 2	0.0001	0.24
206024_at	HPD	4-hydroxyphenylpyruvate dioxygenase	0.0001	0.06
214829_at	AASS	aminoadipate-semialdehyde synthase	0.0001	0.23
204602_at	DKK1	dickkopf homolog 1 (Xenopus laevis)	0.0001	15.29
222771_s_at	MYEF2	myelin expression factor 2	0.0001	3.62
228280_at	ZC3HAV1L	zinc finger CCCH-type, antiviral 1-like	0.0001	3.91
237212_at	---	---	0.0001	0.29
206069_s_at	ACADL	acyl-Coenzyme A dehydrogenase, long chain	0.0001	0.29
230577_at	---	---	0.0001	0.08
210852_s_at	AASS	aminoadipate-semialdehyde synthase	0.0002	0.28
218816_at	LRRC1	leucine rich repeat containing 1	0.0002	4.33
228988_at	ZNF711	zinc finger protein 711	0.0002	4.16
201387_s_at	UCHL1	ubiquitin carboxyl-terminal esterase L1 (ubiquitin thiolesterase)	0.0002	3.36
209260_at	SFN	stratifin	0.0002	4.01
219404_at	EPS8L3	EPS8-like 3	0.0002	3.55
228051_at	LOC202451	Hypothetical protein LOC202451	0.0002	4.73
229700_at	ZNF738	zinc finger protein 738	0.0002	3.82
230602_at	ACMSD	aminocarboxymuconate semialdehyde decarboxylase	0.0002	0.23
244741_s_at	MGC9913	hypothetical protein MGC9913	0.0002	3.15
205222_at	EHHADH	enoyl-Coenzyme A, hydratase/3-hydroxyacyl Coenzyme A deh	0.0003	0.26
205506_at	VIL1	villin 1	0.0003	6.48
214751_at	ZNF468	zinc finger protein 468	0.0003	3.94
222071_s_at	SLCO4C1	solute carrier organic anion transporter family, member 4C1	0.0003	3.67
222108_at	AMIGO2	adhesion molecule with Ig-like domain 2	0.0003	3.81
242496_at	---	---	0.0003	0.20
210652_s_at	TTC39A	tetratricopeptide repeat domain 39A	0.0004	4.14
218847_at	IGF2BP2	insulin-like growth factor 2 mRNA binding protein 2	0.0004	5.49
229614_at	ZNF320	zinc finger protein 320	0.0004	3.33
235144_at	---	---	0.0004	5.15
202934_at	HK2	hexokinase 2	0.0005	3.91
204704_s_at	ALDOB	aldolase B, fructose-bisphosphate	0.0005	0.10
208949_s_at	LGALS3	lectin, galactoside-binding, soluble, 3	0.0005	3.83
228728_at	C7orf58	chromosome 7 open reading frame 58	0.0005	0.33
230030_at	HS6ST2	heparan sulfate 6-O-sulfotransferase 2	0.0005	4.38
231856_at	KIAA1244	KIAA1244	0.0005	3.12
232494_at	CYP8B1	cytochrome P450, family 8, subfamily B, polypeptide 1	0.0005	0.07
241703_at	RUNDC3B	RUN domain containing 3B	0.0005	0.29
206930_at	GLYAT	glycine-N-acyltransferase	0.0006	0.11

208029_s_at	LAPTM4B	lysosomal protein transmembrane 4 beta	0.0006	4.44
213293_s_at	TRIM22	tripartite motif-containing 22	0.0006	0.33
213664_at	SLC1A1	solute carrier family 1 (neuronal/epithelial high affinity glutama	0.0006	0.13
214621_at	GYS2	glycogen synthase 2 (liver)	0.0006	0.09
225681_at	CTHRC1	collagen triple helix repeat containing 1	0.0006	10.27
230554_at	ACSM2A	acyl-CoA synthetase medium-chain family member 2A	0.0006	0.15
232449_at	BCO2	beta-carotene oxygenase 2	0.0006	0.27
243193_at	---	---	0.0006	3.84
1554679_a_at	LAPTM4B	lysosomal protein transmembrane 4 beta	0.0008	6.46
203543_s_at	KLF9	Kruppel-like factor 9	0.0008	0.32
203789_s_at	SEMA3C	sema domain, immunoglobulin domain (Ig), short basic domain,	0.0008	3.48
205352_at	SERPINI1	serpin peptidase inhibitor, clade I (neuroserpin), member 1	0.0008	3.57
208767_s_at	LAPTM4B	lysosomal protein transmembrane 4 beta	0.0008	3.89
208837_at	TMED3	transmembrane emp24 protein transport domain containing 3	0.0008	3.85
215321_at	RUNDC3B	RUN domain containing 3B	0.0008	0.29
216687_x_at	UGT2B15	UDP glucuronosyltransferase 2 family, polypeptide B15	0.0008	0.18
227794_at	GLYATL1	glycine-N-acyltransferase-like 1	0.0008	0.18
228494_at	PPP1R9A	protein phosphatase 1, regulatory (inhibitor) subunit 9A	0.0008	4.82
230931_at	---	---	0.0008	0.28
231790_at	DMGDH	dimethylglycine dehydrogenase	0.0008	0.27
40665_at	FMO3	flavin containing monooxygenase 3	0.0008	0.18
1552568_at	RTP3	receptor (chemosensory) transporter protein 3	0.0010	0.26
205568_at	AQP9	aquaporin 9	0.0010	0.28
207097_s_at	SLC17A2	solute carrier family 17 (sodium phosphate), member 2	0.0010	0.25
209173_at	AGR2	anterior gradient homolog 2 (Xenopus laevis)	0.0010	7.99
211006_s_at	KCNB1	potassium voltage-gated channel, Shab-related subfamily, meml	0.0010	0.19
217127_at	CTH	cystathionase (cystathionine gamma-lyase)	0.0010	0.21
219010_at	C1orf106	chromosome 1 open reading frame 106	0.0010	6.36
222572_at	PDP1	pyruvate dehyrogenase phosphatase catalytic subunit 1	0.0010	3.60
241914_s_at	ACSM2A /// ACSM2B	acyl-CoA synthetase medium-chain family member 2A /// acyl-(	0.0010	0.23
243951_at	ABCB1	ATP-binding cassette, sub-family B (MDR/TAP), member 1	0.0010	0.32
1552569_a_at	RTP3	receptor (chemosensory) transporter protein 3	0.0012	0.15
1552767_a_at	HS6ST2	heparan sulfate 6-O-sulfotransferase 2	0.0012	4.58
201251_at	PKM2	pyruvate kinase, muscle	0.0012	3.26
201428_at	CLDN4	claudin 4	0.0012	3.23
206496_at	FMO3	flavin containing monooxygenase 3	0.0012	0.22
209994_s_at	ABCB1 /// ABC	ATP-binding cassette, sub-family B (MDR/TAP), member 1 ///	0.0012	0.29
214413_at	TAT	Tyrosine aminotransferase	0.0012	0.17
214734_at	EXPH5	exophilin 5	0.0012	0.29
231187_at	SLC28A1	solute carrier family 28 (sodium-coupled nucleoside transporter)	0.0012	0.31
33322_i_at	SFN	stratifin	0.0012	5.74
212092_at	PEG10	paternally expressed 10	0.0015	7.14
203028_s_at	CYBA	cytochrome b-245, alpha polypeptide	0.0015	3.06
206068_s_at	ACADL	acyl-Coenzyme A dehydrogenase, long chain	0.0015	0.25
211357_s_at	ALDOB	aldolase B, fructose-bisphosphate	0.0015	0.26
214069_at	ACSM2A /// AC	acyl-CoA synthetase medium-chain family member 2A /// acyl-(	0.0015	0.26
216379_x_at	CD24	CD24 molecule	0.0015	8.21
228391_at	CYP4V2	cytochrome P450, family 4, subfamily V, polypeptide 2	0.0015	0.29
236652_at	LOC149703	hypothetical protein LOC149703	0.0015	0.25
204694_at	AFP	alpha-fetoprotein	0.0019	7.85
206226_at	HRG	histidine-rich glycoprotein	0.0019	0.21
206913_at	BAAT	bile acid Coenzyme A: amino acid N-acyltransferase (glycine N	0.0019	0.31
207392_x_at	UGT2B15	UDP glucuronosyltransferase 2 family, polypeptide B15	0.0019	0.24
209550_at	NDN	necdin homolog (mouse)	0.0019	4.05
209771_x_at	CD24	CD24 molecule	0.0019	7.62
213920_at	CUX2	cut-like homeobox 2	0.0019	0.19

218051_s_at	NT5DC2	5'-nucleotidase domain containing 2	0.0019	3.82
222943_at	GBA3	glucosidase, beta, acid 3 (cytosolic)	0.0019	0.16
231691_at	C3P1	complement component 3 precursor pseudogene	0.0019	0.23
232428_at	MOGAT2	monoacylglycerol O-acyltransferase 2	0.0019	0.22
233587_s_at	SIPA1L2	signal-induced proliferation-associated 1 like 2	0.0019	4.03
33323_r_at	SFN	stratifin	0.0019	6.65
204439_at	IFI44L	interferon-induced protein 44-like	0.0024	0.31
206170_at	ADRB2	adrenergic, beta-2-, receptor, surface	0.0024	0.31
206916_x_at	TAT	tyrosine aminotransferase	0.0024	0.31
207201_s_at	SLC22A1	solute carrier family 22 (organic cation transporter), member 1	0.0024	0.06
218454_at	PLBD1	phospholipase B domain containing 1	0.0024	3.09
219580_s_at	TMC5	transmembrane channel-like 5	0.0024	3.87
220017_x_at	CYP2C9	cytochrome P450, family 2, subfamily C, polypeptide 9	0.0024	0.19
225081_s_at	CDCA7L	cell division cycle associated 7-like	0.0024	3.01
225316_at	MFSD2	major facilitator superfamily domain containing 2	0.0024	0.20
226096_at	FNDC5	fibronectin type III domain containing 5	0.0024	0.18
229019_at	ZNF385B	zinc finger protein 385B	0.0024	0.29
235700_at	CT45A5	cancer/testis antigen family 45, member A5	0.0024	3.09
31835_at	HRG	histidine-rich glycoprotein	0.0024	0.22
1553986_at	RASEF	RAS and EF-hand domain containing	0.0029	3.26
201416_at	SOX4	SRY (sex determining region Y)-box 4	0.0029	3.78
204428_s_at	LCAT	lecithin-cholesterol acyltransferase	0.0029	0.29
205866_at	FCN3	ficolin (collagen/fibrinogen domain containing) 3 (Hakata antig	0.0029	0.26
206797_at	NAT2	N-acetyltransferase 2 (arylamine N-acetyltransferase)	0.0029	0.21
208650_s_at	CD24	CD24 molecule	0.0029	8.85
208651_x_at	CD24	CD24 molecule	0.0029	7.02
209656_s_at	TMEM47	transmembrane protein 47	0.0029	0.29
209772_s_at	CD24	CD24 molecule	0.0029	4.50
211756_at	PTHLH	parathyroid hormone-like hormone	0.0029	3.12
214421_x_at	CYP2C9	cytochrome P450, family 2, subfamily C, polypeptide 9	0.0029	0.17
219733_s_at	SLC27A5	solute carrier family 27 (fatty acid transporter), member 5	0.0029	0.24
227194_at	FAM3B	family with sequence similarity 3, member B	0.0029	5.23
231683_at	GLYAT	glycine-N-acyltransferase	0.0029	0.25
204705_x_at	ALDOB	aldolase B, fructose-bisphosphate	0.0035	0.28
207086_x_at	GAGE1 /// GAC	G antigen 1 /// G antigen 12C /// G antigen 12D /// G antigen 12	0.0035	8.07
207256_at	MBL2	mannose-binding lectin (protein C) 2, soluble (opsonic defect)	0.0035	0.33
207608_x_at	CYP1A2	cytochrome P450, family 1, subfamily A, polypeptide 2	0.0035	0.32
209612_s_at	ADH1B	alcohol dehydrogenase 1B (class I), beta polypeptide	0.0035	0.32
209613_s_at	ADH1B	alcohol dehydrogenase 1B (class I), beta polypeptide	0.0035	0.27
209614_at	ADH1B	alcohol dehydrogenase 1B (class I), beta polypeptide	0.0035	0.15
210168_at	C6	complement component 6	0.0035	0.22
218312_s_at	ZSCAN18	zinc finger and SCAN domain containing 18	0.0035	3.26
219954_s_at	GBA3	glucosidase, beta, acid 3 (cytosolic)	0.0035	0.21
226051_at	SELM	selenoprotein M	0.0035	3.84
240304_s_at	TMC5	transmembrane channel-like 5	0.0035	6.17
203214_x_at	CDC2	cell division cycle 2, G1 to S and G2 to M	0.0043	3.08
204580_at	MMP12	matrix metallopeptidase 12 (macrophage elastase)	0.0043	3.83
205476_at	CCL20	chemokine (C-C motif) ligand 20	0.0043	3.98
205554_s_at	DNASE1L3	deoxyribonuclease I-like 3	0.0043	0.25
216025_x_at	CYP2C9	cytochrome P450, family 2, subfamily C, polypeptide 9	0.0043	0.19
225056_at	SIPA1L2	signal-induced proliferation-associated 1 like 2	0.0043	4.28
230316_at	SEC14L2	SEC14-like 2 (S. cerevisiae)	0.0043	0.27
266_s_at	CD24	CD24 molecule	0.0043	7.78

NOTE: HCV, hepatitis C virus

TABLE-S4. The candidate genes were associated with poorly differentiated HCCs whose hepatic backgrounds were associated with HBV or HCV

Probe set	Symbol	Title	well vs. poor in HBV(+)		well vs. poor in HCV(+)	
			p-value	Fold-change	p-value	Fold-change
204351_at	S100P	S100 calcium binding protein P	0.0012	22.46	<0.0001	32.93
206574_s_at	PTP4A3	protein tyrosine phosphatase type IVA, member 3	0.0012	3.14	<0.0001	3.42
206916_x_at	TAT	tyrosine aminotransferase	0.0022	0.27	0.0024	0.31
204580_at	MMP12	matrix metallopeptidase 12 (macrophage elastase)	0.0037	3.21	0.0043	3.83
214413_at	TAT	Tyrosine aminotransferase	0.0037	0.16	0.0012	0.17

NOTE: HBV, hepatitis B virus; HCV, hepatitis C virus

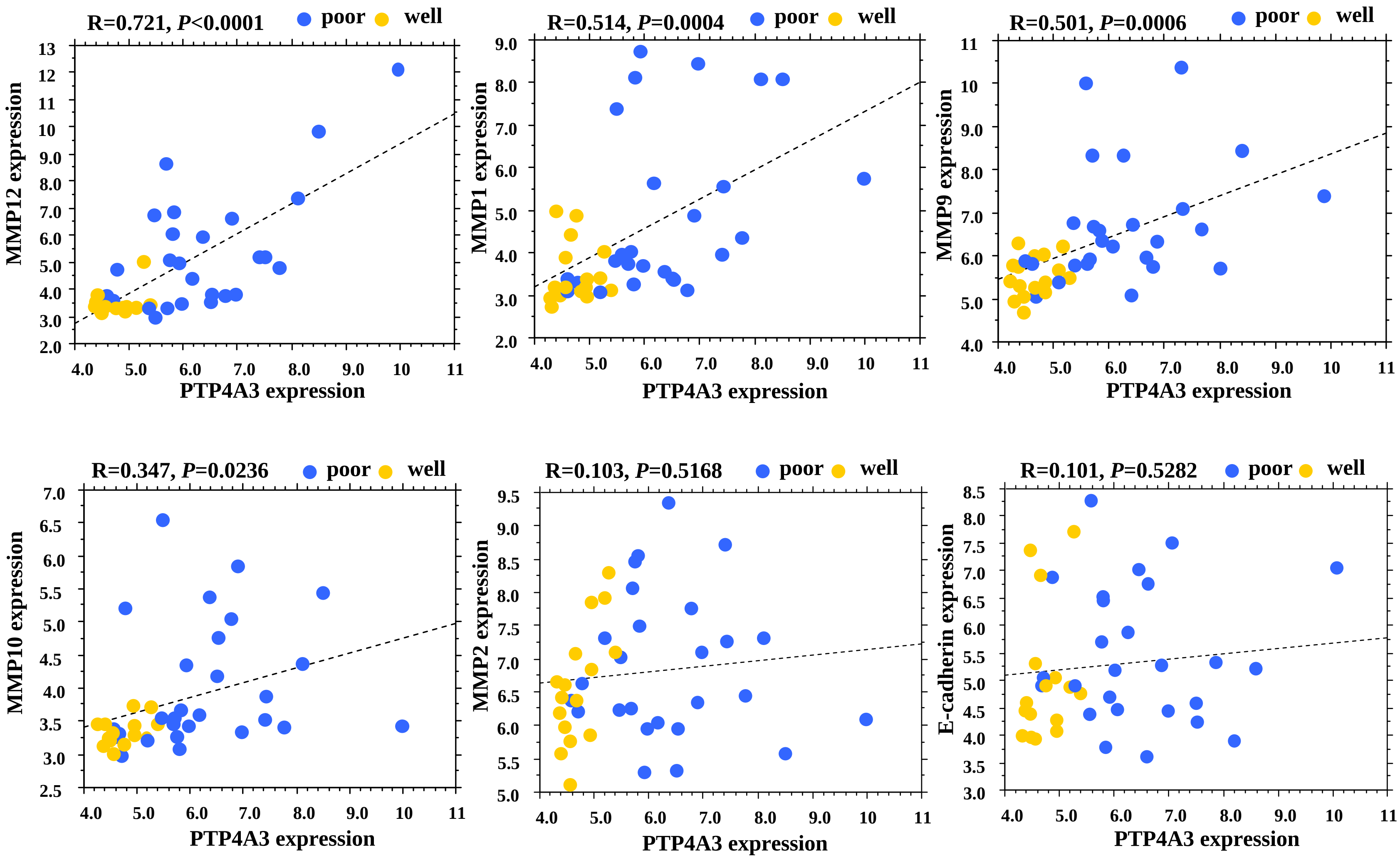

Fig. S1

Abudureheman Mayinuer
